# Enterovirus D68 Subgenotype B3 Circulation in Children with Acute Respiratory Illness in the State of Alagoas, Brazil

**DOI:** 10.3390/v17020242

**Published:** 2025-02-11

**Authors:** Alex Ranieri Jerônimo Lima, Hazerral de Oliveira Santos, James Siqueira Pereira, Anderson Brandão Leite, Jean Phellipe Marques do Nascimento, Juliana Vanessa Cavalcante Souza, Marlon Breno Zampieri Lima, Mykaella Andrade de Araújo, Marta Giovanetti, Esper Georges Kallas, Sandra Coccuzzo Sampaio, Maria Carolina Elias, Svetoslav Nanev Slavov

**Affiliations:** 1 Center for Viral Surveillance and Serologic Evaluation (CeVIVAs), Butantan Institute, Av. Vital Brasil 1500, São Paulo CEP 05585-000, Brazil; alex.lima@fundacaobutantan.org.br (A.R.J.L.); james.pereira.esib@esib.butantan.gov.br (J.S.P.); esper.kallas@butantan.gov.br (E.G.K.); sandra.coccuzzo@butantan.gov.br (S.C.S.); carolina.eliassabbaga@butantan.gov.br (M.C.E.); 2 Programa de Interunidades de Pós-Graduação em Bioinformática, Universidade de São Paulo, Av. Lineu Prestes 748, São Paulo CEP 05508-900, Brazil; 3 Central Laboratory of Public Health (LACEN)-Alagoas, Rua Ernesto Gomes Maranhão 1773, Maceio CEP 57036-860, Brazil; hazerral@hotmail.com (H.d.O.S.); anderson.leite@icbs.ufal.br (A.B.L.); jeanphellipe.biologo@gmail.com (J.P.M.d.N.); ju.biomedica@hotmail.com (J.V.C.S.); mykaella.araujo@saude.al.gov.br (M.A.d.A.); 4 Post-Graduation Program in Clinical Oncology, Stem Cells and Cell Therapy, Faculty of Medicine of Ribeirão Preto, University of São Paulo-USP, Av. Bandeirantes 3900, Ribeirão Preto CEP 14049-900, Brazil; marlon.zlima@usp.br; 5 Sciences and Technologies for Sustainable Development and One Health, Università Campus Bio-Medico di Roma, Via Alvaro del Portillo 21, 00128 Roma, Italy; giovanetti.marta@gmail.com; 6 Instituto Rene Rachou, Fundação Oswaldo Cruz, Av. Augusto de Lima 1715, Belo Horizonte CEP 30190-002, Brazil

**Keywords:** Enterovirus D68, EV-D68, genotype B, pediatric patients, phylogeny, Brazil

## Abstract

Enterovirus D68 (EV-D68) is a leading cause of acute respiratory disease outbreaks, especially among children. EV-D68 infections can rapidly progress to severe clinical complications and potentially fatal outcomes. In Brazil, no diagnostic or genomic surveillance of this virus is currently performed. Between July and September 2023, cases of acute EV-D68 infection were identified among pediatric patients in several municipalities within the State of Alagoas, Northeast Brazil. Infections were confirmed by RT-qPCR using nasopharyngeal samples, and the complete EV-D68 genomes were sequenced and analyzed through phylogenetic inference. EV-D68 RNA was identified in four children aged 1–9 years from four geographically distinct municipalities in Alagoas. All infections were associated with lower respiratory tract symptoms, including dyspnea and wheezing; however, no fatalities were reported. Complete genomic sequencing revealed that the samples belonged to genotype B, subgenotype B3. This is the first study to report complete genomic data on EV-D68 infections from Brazil and South America. Enhanced genomic surveillance and focused EV-D68 diagnosis are critical to better understanding and managing the regional and national dissemination of this virus.

## 1. Introduction

Enterovirus D68 (EV-D68), a member of the genus *Enterovirus* (Species *Enterovirus deconjuncti*) in the *Picornaviridae* family [1], causes a pediatric respiratory infection, ranging from mild to severe. Symptoms may include fever, sore throat, cough, and congestion, with dyspnea and wheezing being notable findings [2]. In severe cases, EV-D68 can progress to the lower respiratory tract and cause neurological complications such as meningitis, encephalitis, and acute flaccid paralysis [3,4]. Phylogenetically, EV-D68 has been classified into four main genotypes, labeled from A to D, with subgenotypes (clades) B3 and A2/D2 being the most prevalent worldwide [5].

The surveillance and systematic detection of EV-D68 in Brazil have not been established. Several studies on the detection of non-polio enteroviruses in Brazil between 2001 and 2021 have reported a predominance of EV-B group enteroviruses, followed by representatives of the EV-A and EV-C groups, respectively. Although uncommon enteroviruses such as EV-A71, CVB2, E11, EV-99, CVA11, and CVA12 have been identified, EV-D68 and other enteroviruses from the EV-D group have never been systematically detected [6,7,8]. Consequently, the extent of EV-D68 dissemination and its clinical impact remains largely unknown in Brazil, the largest country in Latin America. Since 2015, only isolated case reports of EV-D68 have been documented [9,10,11]. To date, no complete EV-D68 genomes from Brazil or South America have been made available.

In a previous viral metagenomic study [12], we identified a high number of EV-D68 sequence reads in several patients who tested negative for the viral respiratory panel provided by the Brazilian Ministry of Health. This raised important questions regarding the molecular and genotypic characteristics of EV-D68 in these cases. Consequently, we confirmed the presence of EV-D68 RNA in individual nasopharyngeal samples using in-house RT-qPCR, sequenced the complete EV-D68 genomes, and performed phylogenetic analyses. This is the first report describing the genotypes of EV-D68 circulating in the State of Alagoas and Brazil and it provides the first complete genomes of this viral agent in South America.

## 2. Materials and Methods

### 2.1. Clinical Samples

**Figure 1:**
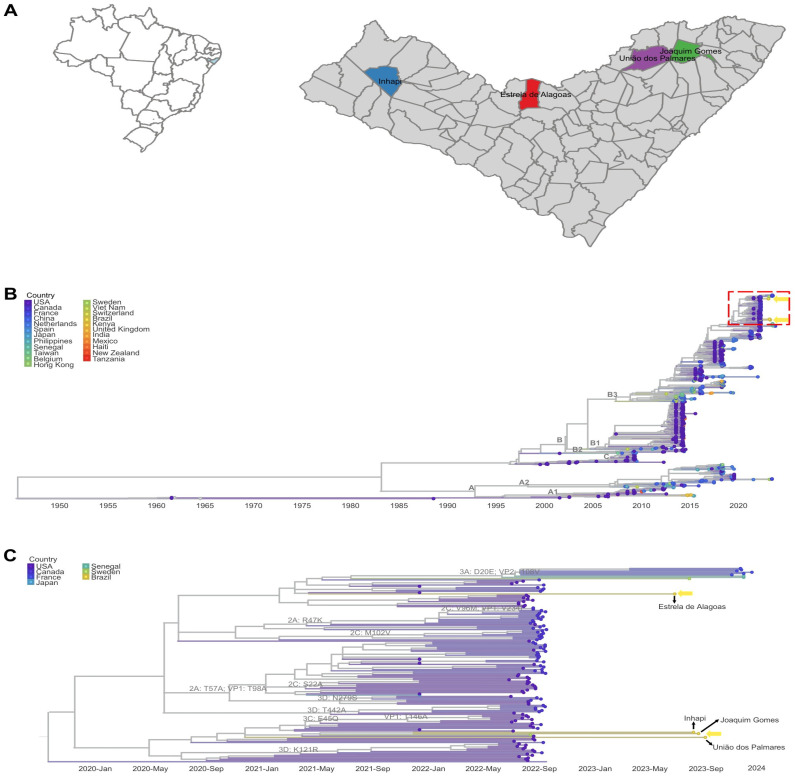
Spatial area of the study and phylogenetic analysis of EV-D68 circulating in the State of Alagoas, Brazil. (**A**) Map of Brazil highlighting the northeastern state of Alagoas, with colored municipalities indicating the locations where positive cases of EV-D68 were identified. (**B**) Maximum-likelihood phylogenetic tree, reconstructed from 1077 complete EV-D68 reference genomes, alongside four sequences obtained in this study. The phylogeny was inferred using the TIM+F+I+G4 substitution model, with branch support provided by 5000 ultrafast bootstrap replicates. (**C**) A focused view of the B3 subgenotype cluster is shown, where the Brazilian sequences are marked according to their geographic origin within the State of Alagoas. The complete EV-D68 sequences have been deposited in the NCBI GenBank (accession numbers: PQ432995–PQ432998).

This study originated from a broader metagenomic study aimed at investigating the etiological diagnosis of acute respiratory symptoms among patients with negative results for SARS-CoV-2, influenza A, influenza B, metapneumovirus, respiratory syncytial virus, adenovirus, and rhinovirus/enterovirus via the routinely used diagnostic tests: the fiveplex kit for SARS-CoV-2/influenza A/influenza B/metapneumovirus/RNase P and the fourplex kit for respiratory syncytial virus/adenovirus/rhinovirus/RNase P supplied by the Brazilian Ministry of Health (Bio-Manguinhos, Rio de Janeiro, Brazil). This initial study revealed four samples that presented high quantities of EV-D68 reads. These samples belonged to four children from different municipalities of the State of Alagoas (Figure 1A). This study was approved by the Institutional Ethics Committee of the Faculty of Medicine of Sao Paulo, University of Sao Paulo (approval number CAAE 68586623.0.0000.0068).

### 2.2. Enterovirus D68 RNA Extraction and RT-qPCR Detection

Extraction of viral RNA was performed manually from 140 µL nasopharyngeal swabs with the Qiagen Viral Nucleic Acids Mini Kit (QIAGEN, Hilden, Germany), following the manufacturer’s instructions. Confirmation of EV-D68 RNA was performed via in-house-developed RT-qPCR targeting the 5′UTR region of the EV-D68 genome. In the reaction, forward EV-D68F (5′-TGCGGCTAATCCTAACCATG-3′) and reverse EV-D68R (5′-AACACGGACACCCAAAGTAG-3′) primers and the hydrolytic probe EV-D68P (FAM-5′ TTACGACAAGCAACTCACTGGCCT-3′-TAMRA) were applied in concentrations of 400 nM for the primers and 250 nM for the probe, respectively. The reaction was performed using the 1X GoTaq 1-Step RT-qPCR system (Promega, Madison, WI, USA) in a final volume of 20 μL. Amplification was performed on a 7500 Real-Time system (ThermoFisher, Waltham, MA, USA) under the cycling conditions including reverse transcription at 40 °C for 40 min, denaturation at 95 °C for 2 min, and 40 cycles at 95 °C for 15 s and 60 °C for 1 min.

The complete EV-D68 genome was sequenced using in-house primers designed using the Primal Scheme software v3.0.2 (https://primalscheme.com, accessed on 30 April 2024; see Supplementary Table S1). For the design of the primers, a multiple alignment employing the most common EV-D68 genotypes was applied with complete genomes originating from the USA, France, Mexico, Japan, Haiti, China, Taiwan, and India. Library preparation followed Illumina’s COVIDSeq Test Kit protocol (Illumina, San Diego, CA, USA), substituting SARS-CoV-2 primers with the EV-D68 primers for complete genome sequencing. The amplified DNA was fragmented and indexed, and the pooled libraries were sequenced on the Illumina MiSeq2 system using the MiSeq2 Nano Kit v2 flow cell (paired-end 150).

### 2.3. Genomic Assembly

Initially, primers and adapters were removed from the raw reads using Cutadapt v2.8 [13] with default parameters. The reads were then processed with Trimmomatic [14] to eliminate low-quality sequences, applying the parameters LEADING:3, TRAILING:3, SLIDINGWINDOW:5:20, and MINLEN:35. Subsequently, for each sample, the filtered reads were utilized to select the optimal reference genome using VAPOR [15] with default settings. This process employed an EV-D68 genome database comprising 1077 sequences obtained from human hosts, which was downloaded from NCBI Virus on 3 July 2024. The filtered reads were mapped against the selected reference genome using Bowtie2 v2.5.4 [16] with default parameters. A BAM file was generated using SAMtools v 1.21 [17] to serve as the input for Pilon [18], with the parameters -mindepth 5 and -minmq 10, to correct small gaps and indels.

To further refine the read mapping and obtain the final genome consensus, the FASTA sequence generated by Pilon was used as a reference for mapping the filtered reads using Bowtie2. The consensus genome was then obtained using iVar [19] with the parameters -q 20 and -m 5. To evaluate the quality of the assembly and derive assembly statistics, including the number of mapped reads and sequencing depth, an additional round of mapping was conducted to align the reads with the final genome consensus. From this final mapping, SNP detection was performed using iVar, considering only sites with a minimum sequencing depth of 100 to call SNPs with a minimum frequency of 3%.

The iVar output was used to count the number of intrahost single nucleotide variants (iSNVs) for each gene per nucleotide site, excluding indels and assembly gap (N) sites. Only significant SNP calls (*p*-value < 0.05) were considered.

### 2.4. Phylogenetic Analysis

The four genomes assembled in this study, along with 1077 genomic sequences downloaded from NCBI Virus, were utilized for phylogenetic analysis. The dataset was aligned using the Augur align tool within the Augur program toolkit v24.1.0 [20], employing the MAFFT v.7.520 algorithm [21]. Subsequently, a maximum likelihood (ML) phylogenetic tree was constructed using IQ-TREE v2.2.6 [22]. The tree was reconstructed with the TIM+F+I+G4 nucleotide substitution model, selected by the ModelFinder application [23] within IQ-TREE2. To assess the robustness of the tree topology, 5000 UFBoot replicates were generated. The phylogenetic tree was dated using Treetime v0.11.2 [24], integrated into Augur. Multiple refinements were applied during this phase, including rerooting the tree using the midpoint method, inferring node confidence dates, and stochastically resolving polytomies.

Ancestral inference and identification of nucleotide mutations were conducted using Augur ancestral, while amino acid mutations were annotated with Augur translate, referencing the EV-D68 Fermon strain (GenBank accession AY426531.1). Clades were annotated as previously defined by Hodcroft et al. (2022) [25], utilizing the clades file provided on GitHub (https://github.com/nextstrain/enterovirus_d68, accessed on 8 August 2024).

## 3. Results

The children found to be positive for EV-D68 RNA were between 1 and 9 years of age (mean 5.5 years of age) and the samples were obtained between July and September 2023 from four distantly located municipalities of the State of Alagoas (Figure 1A). All patients presented acute infection characterized by dyspnea and wheezing. Other symptoms like fever, cough, vomiting, coryza, nasal congestion, and oxygen saturation <95% were also registered. No lethality was observed. One of the patients had asthma as a comorbidity. EV-D68 was confirmed by RT-qPCR in all clinical samples. The mean cycle threshold (Ct) of acute infection was 27.46 (range: 25.47–29.95).

The phylogenetic analysis revealed that all genomic sequences obtained in this study belonged to the major genotype B, subgenotype B3 (Figure 1B). All Brazilian sequences formed a monophyletic cluster, which also comprised genome sequences isolated in the USA. This cluster was further subdivided into two subclusters. One of our sequences clustered with strains from the USA, Sweden, and France, while the other three sequences were grouped in a subcluster consisting entirely of strains isolated in the USA in 2022.

## 4. Discussion

EV-D68 has emerged as a significant public health problem due to its severe clinical manifestations, especially in the pediatric population. Until recently, EV-D68 outbreaks were reported only sporadically. However, during the COVID-19 pandemic, the circulation of EV-D68 was likely interrupted due to the public health measures implemented to mitigate viral transmission. With the subsequent relaxation of these measures, there has been a substantial resurgence in EV-D68 infections, leading to extensive outbreaks. Notably, this increased circulation was observed in Europe in 2021 and culminated in a large outbreak among pediatric patients in the United States [26].

A major challenge faced in Brazil is the lack of information regarding the extent of EV-D68 circulation and genotype dissemination within the country. The absence of a specific EV-D68 diagnostic test complicates the notification and reporting of this infection. In this line, the diagnosis of the EV-D68 cases in our study and their genomic sequencing was only made possible after the application of viral metagenomics in acute respiratory infection cases without an etiological diagnosis that revealed sequence reads specifically for this virus. In light of this, our study provides the first complete EV-D68 genome characterization from both Brazil and South America. The phylogenetic analysis revealed the circulation of the EV-D68 genotype B, subgenotype B3, which shows worldwide dissemination [5]. Three of our sequences clustered within a monophyletic group predominantly composed of complete EV-D68 genomes circulating in the United States mainly in 2022. Another sequence was grouped within a separate cluster, containing isolates from the United States, Canada, France, Senegal, and Sweden that circulated between 2021 and 2023. This sequence was grouped with an isolate from the United States, and this multi-country cluster has an ancestral node that connects to a monophyletic cluster of isolates from the United States. This could suggest the introduction of the Brazilian EV-D68 isolates from the United States into the State of Alagoas, a well-known tourist destination. Alternatively, it may represent a genotype already circulating in the Americas, including Brazil, as it is one of the most globally widespread genotypes. Given the rapid evolution of EV-D68 [25], continuous genomic surveillance and the investigation of suspected outbreaks are crucial. A notable finding in our study is that samples positive for EV-D68 initially tested negative for rhinovirus/enterovirus using the routine kit employed by the Brazilian Ministry of Health. This underscores the need for improved diagnostic methods with more specific EV-D68 detection systems and virus genotyping [27].

The EV-D68 positivity and complete genomes obtained in this study probably largely underestimate the true extent of the prevalence and frequency of this genotype in Brazil, considering the country’s vast territory, diverse demographics, and varied urban settings. Another important question is whether the obtained genomes belonged to an EV-D68 outbreak in the State of Alagoas or whether they only represented sporadic cases. The period of sample collection (July–September) coincided with the period of EV-D68 outbreaks in the Northern Hemisphere [27,28], i.e., summer to fall (Brazilian winter), suggesting that EV-D68 might show a very similar pattern of circulation. Therefore, the observed cases might be part of an EV-D68 outbreak that hit Northeast Brazil. However, due to the insufficient detection and surveillance of EV-D68 in Brazil, we are unable to gather adequate information regarding its temporal characteristics or magnitude. Additionally, the positive samples were detected only in four northern municipalities, without giving a complete picture of the whole state. For this reason, further investigations in a larger subset of acute respiratory samples without etiological diagnosis collected throughout the state’s territory are needed to determine the extent of EV-D68 infections in this location.

In conclusion, this study is the first in Brazil and South America to characterize the genotypic features of EV-D68 infection. Enhanced genomic surveillance and more comprehensive diagnostic measures are essential for better understanding and managing the spread of this virus in the region.

## Data Availability

The complete EV-D68 genomes were deposited in the NCBI GenBank under the following accession numbers: PQ432995–PQ432998.

## Supplementary Materials

The following supporting information can be downloaded at: https://www.mdpi.com/article/10.3390/v17020242/s1, Table S1: Primer sets for complete genomic sequencing of EV-D68.
