# Supplementary material for: Enterovirus D68 Subgenotype B3 Circulation in Children with Acute Respiratory Illness in the State of Alagoas, Brazil
Source: Viruses. 2025 Feb 11;17(2):242. doi: 10.3390/v17020242 (PMC11860543; doi:10.3390/v17020242)
Supplement: Supplementary file 1 [file viruses-17-00242-s001.zip › viruses-3439704-supplementary.pdf]

Table S1. Used primer sets for the complete genomic sequencing of Enterovirus D68.

D68_1_L	CTTTGTACGCCTGTTTTATCTCCC
D68_1_R	TTAGGATTAGCCGCATTCAGGG
D68_2_L	GCGGCCTACTCATGGTGAAA
D68_2_R	TGTGATATGAGATCCATTTGTGGCA
D68_3_L	GACTACTTTGGGTGTCCGTGTT
D68_3_R	CTCTATCACTGTAGCCACACGC
D68_4_L	CAGGACCCATCGAAATTCACTGA
D68_4_R	TTTGTGGCATTACACTGCACATG
D68_5_L	TGTGCAGTGTAATGCCACAAAATT
D68_5_R	GCGTGTCTGAGTCCATTAAACTC
D68_6_L	GGATGAATGCTGCTCCAATGGA
D68_6_R	TCTGTCAATGCTGATATGTCAACCT
D68_7_L	ATTCCAGGGCAGGTCCGTAA
D68_7_R	CCACTAATCCAAGGTATTATCAGGGT
D68_8_L	ACAACCAGAGAGACCGCCAT
D68_8_R	GTTTCAACTGCATTTAAGCTAGGGAC
D68_9_L	AGGGTTCATAGCAGCAAAAGATGA
D68_9_R	AAGGACCTGGTGTTAATTGTCCA
D68_10_L	ACAAACTCGCACAGTGATAAATCA
D68_10_R	GGGGGTCAGAGATTTTAAAGAATACAC
D68_11_L	TGGGTCTTCCTGACTTGACACT
D68_11_R	GTGCTCTTTCTTTACCTTTGTAATTTGC
D68_12_L	CCTAAACACATAAAAGCGTGGGC
D68_12_R	CCCTGGGCCTTCAAAGCAAA
D68_13_L	AGACAGTGTTAAAACCATGCCTCA
D68_13_R	ACCCCCTCCTGCTGTAAGAA
D68_14_L	TCGGTCTTCTTACAGCAGGAGG
D68_14_R	AGCCACGATTCACTCTGTCTAG
D68_15_L	AATGCCTTTGGAGCAGGGTT

D68_15_R	TTCTCCCTAGCTTCCGGTAATATTTT
D68_16_L	CATGCAATGCTCTCAGAGGTCT
D68_16_R	TCTGTGATAGCCCTGGCAATTAAA
D68_17_L	TCCAGTTCAAGTCCAAATCTCGC
D68_17_R	AACCTGCGTGACAAAGCCTT
D68_18_L	ACTCAAAGGCTTTGTCACGCA
D68_18_R	GGCAATAATCCCTAACTTCTTGAGAATC
D68_19_L	GGAAAGGCTATTCAATTCAGAGATCG
D68_19_R	GCCCTGAATGCCAGCAAAAA
D68_20_L	CTAAAGTGCAAGGACCAGGGTT
D68_20_R	CCACCTAGGTTCAAGAAGCCAT
D68_21_L	ATCCCCACACACGCATCTGT
D68_21_R	GCTGCAAATCCTTGAGCTCCAT
D68_22_L	AACAAGAGCTGGCCAGTGTG
D68_22_R	AGGGGTATGGGATCCACACT
D68_23_L	AAGGAGCCAGCAGTTCTCAAT
D68_23_R	GCGTGATTTCCCTTTTTCAACTTTTT
D68_24_L	AGATCAAGAGAAAAAGTTGAAAAAGGGAA
D68_24_R	TGAATATGCTGGTACCTGAAGAACC
